# Veterinary Education during Covid-19 and Beyond—Challenges and Mitigating Approaches

**DOI:** 10.3390/ani11061818

**Published:** 2021-06-18

**Authors:** Jennifer Routh, Sharmini Julita Paramasivam, Peter Cockcroft, Vishna Devi Nadarajah, Kamalan Jeevaratnam

**Affiliations:** 1 School of Veterinary Medicine, University of Surrey, Vet School Main Building, Daphne Jackson Road, Guildford GU2 7AL, UK; s.paramasivam@surrey.ac.uk (S.J.P.); p.cockcroft@surrey.ac.uk (P.C.); drkamalanjeeva@gmail.com (K.J.); 2 Division of Human Biology, School of Medicine and IMU Centre for Education, International Medical University, No.126, Jalan Jalil Perkasa 19, Bukit Jalil, Kuala Lumpur 57000, Malaysia; vishnadevi_nadarajah@imu.edu.my

**Keywords:** Covid-19, curriculum, e-learning, educational methods

## Abstract

**Simple Summary:**

The Covid-19 pandemic has significantly impacted the provision of veterinary education. The principal challenges and mitigating approaches have focussed on the delivery of material online, but given the practical and professional outcomes required of veterinary graduates, innovative ways to provide some in person teaching have been sought also.

**Abstract:**

The public health implications of the Covid-19 pandemic have caused unprecedented and unexpected challenges for veterinary schools worldwide. They are grappling with a wide range of issues to ensure that students can be trained and assessed appropriately, despite the international, national, and local restrictions placed on them. Moving the delivery of knowledge content largely online will have had a positive and/or negative impact on veterinary student learning gain which is yet to be clarified. Workplace learning is particularly problematic in the current climate, which is concerning for graduates who need to develop, and then demonstrate, practical core competences. Means to optimise the learning outcomes in a hybrid model of curriculum delivery are suggested. Specific approaches could include the use of video, group discussion, simulation and role play, peer to peer and interprofessional education.

## 1. Introduction

In the final week of December 2019, the Hubei Integrated Chinese and Western Medicine hospital in Wuhan reported a clustered point-source outbreak of pneumonia [1], of unknown viral origin. Within 30 days, the rapid geographic spread of the disease, which the International Committee on Taxonomy of Viruses later coined Coronavirus Disease 2019 (Covid-19) [2], implied propagation by human-to-human transmission. On March 11th 2020, the World Health Organization (WHO) designated Covid-19 a pandemic [3]. Covid-19 has been confirmed as the cause of over 130,000,000 cases and over two and a half million deaths globally [4].

However, what began as a ‘healthcare crisis’ quickly had serious implications for all public institutions including universities and veterinary schools in the United Kingdom (UK). National regulations required that students were sent home prior to ‘lockdown’ with little or no advanced warning and caused major disruptions to the delivery of veterinary programmes at all levels.

Historically, and until very recently, veterinary education in the UK has largely relied on the integration of didactic face-to-face teaching with experiential, hands on workplace learning, both of which had to be transformed rapidly. Immediate innovation was required by our veterinary schools to balance their responsibilities regarding the enforcement of public health regulations whilst ensuring that students continue to receive the necessary training based on the professional registration board competencies and standards.

In this article, we discuss some of the problems faced by veterinary educators in the wake of the global pandemic. We describe the specific mitigating approaches which have been implemented already and, drawing on the medical education field, provide examples of new ideas which could be utilised during and beyond the pandemic. This review is by no means exhaustive, and it may not reflect all approaches taken by all veterinary schools. Additionally, the field of health professions education is dynamic. More pedagogical methods and materials are constantly being developed, which may be absent here. 

## 2. Theoretical Content Delivery 

### 2.1. The Problems

One of the most immediate changes introduced at the beginning of the pandemic was the cancellation of all face-to-face teaching and its replacement with online content. There are prominent differences between preparing to teach in the classroom and online. Online education is usually curated and developed using the ADDIE model [5] (analyse, design, develop, implement, and evaluate) which takes a significant amount of time. However, given the immediacy of the challenge during the pandemic, a more ‘cut and paste’ approach for providing content to students in the short term could have been employed. The precise impact of these changes on veterinary students’ experiences and learning gain is yet to be determined, but a questionnaire of 1392 veterinary students from 92 countries demonstrated that over 95% of students said that the pandemic had affected their study or research in some capacity and 47.5% said ‘greatly’ [6].

Despite the taught content being largely identical, there is a loss of the collaborative experience for students when teaching goes online [7]. This is a significant drawback with the benefits of attending classes in person such as real time feedback and sense of community largely absent. Students needed time to transition to online learning, gain the discipline to work to a home-based schedule and avoid screen fatigue.

Barriers to online learning require some consideration. Some obstacles, such as institutional ‘cultural resistance’, are less pertinent in the current climate given that there is an absolute necessity to shift to online provision of education. Nevertheless, for some staff and students, there have been computer skill deficits and/or software unfamiliarity which have needed to be tackled promptly [8], with extra time required for specific training and workshops to improve e-literacy. Faculties need to make sure that the extra workload educators shoulder in order to familiarise themselves with, and use, these online tools is monitored and, in an ideal world, properly compensated for. In the current climate, time management could be challenging for educators with such teaching commitments, with research, clinical work, and life outside of work all possibly demanding more time than normal. The latter is particularly important; extra childcare and other family needs may require flexibility in work patterns.

More globally, technology could be another barrier to online learning. In low-medium income countries, poor infrastructure, including internet connectivity and access to computers, will cause problems for both virtual educators and online remote student learners [6,9], which could be compounded by poor institutional support and lack of communication [10]. In high income countries, not all wi-fi has equivalent speed and stability, and poor connectivity causes problems for students and staff engaging with ‘live’ teaching. The software that provides the platform from which online teaching takes place represent an additional cost to institutions. Additionally, with the restricted access to campus internet and computers, students are mostly dependent on their own devices. Although it may be reasonable to expect the majority of learners to have at least one type of device [11], this does not necessarily equate to all being suitable for use. Finally, even having access to the appropriate technology does not guarantee that the user is able to use it effectively. Some participants are sharing devices with or facing interruption from other members of their household.

### 2.2. The Mitigating Approaches

#### 2.2.1. Practical Aspects

Veterinary schools will be following overarching advice from their relevant government agencies (e.g., Public Health England), which could limit the scope for inventiveness in education provision. Guidance and regulation have driven educators down a virtual route for the 2020/21 academic year, but most schools have been able to offer some form of hybrid/blended courses: teaching online with a degree of socially distanced face-to-face teaching where it is necessary.

Some departments already have the infrastructure in place to adapt to these changes quickly. Most veterinary schools have online learning management software for uploading material (such as pre-recorded lectures), which are likely to be familiar to some faculty staff. Compared to clinical training, pre-clinical (basic sciences) syllabi tend to be delivered in a more systematic fashion, which could make online material easier to construct and organise online for these subjects. There is also scope for interprofessional collaboration with medical/nursing/pure sciences departments who are all teaching disciplines, such as anatomy, physiology, and pharmacology. This increases the demand for this material and could distribute the load for its production.

**Figure 1:**
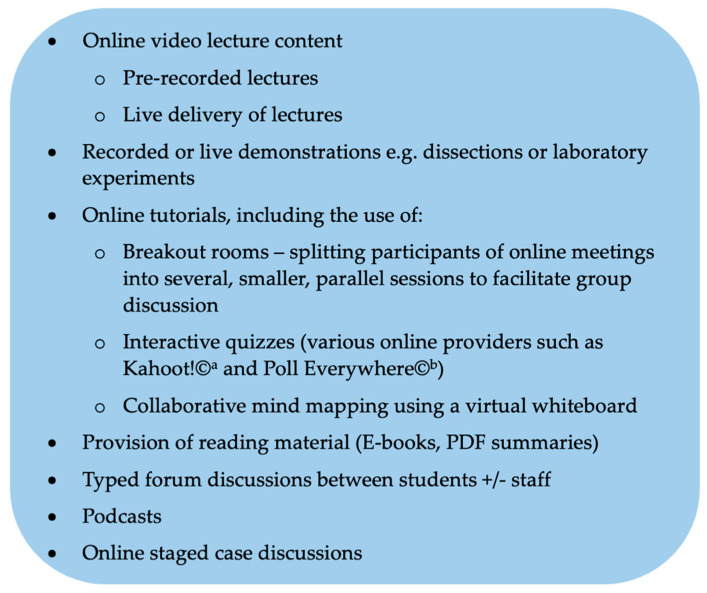
A non-exhaustive list of options for delivering theoretical content online. Kahoot!©-Tordenskiolds gate 6, 0160 Oslo, Norway. https://kahoot.com (accessed on 17 June 2021). Poll Everywhere Inc©-639 Howard Street, San Francisco, CA 94105 USA. https://polleverywhere.com (accessed on 17 June 2021).

However, relying solely on recording those lectures on the existing timetable alongside signposts to self-directed learning resources are not adequate for student engagement. Several options for delivery of content online are listed in Figure 1. Their appropriateness and acceptance by students and educators will depend on the subject of interest and the infrastructure available. The parasitology teaching group at Melbourne Veterinary School have reported positive experiences with the rapid adoption and improvisation of online parasitology teaching during the pandemic [12]. They have utilised some pre-recorded lectures (with the benefit of avoiding network issues during live streaming), complemented by a flipped classroom approach using audiovisual conferencing and student polling. Reported benefits of the flipped classroom include improved engagement, a better use of time and enhanced student-faculty contact, all of which are particularly attractive in the face of the pandemic [13]. The group report that pre- and post- pandemic academic results were comparable.

The notion of delivering content almost completely online is daunting, but when considered in the context of the generation being educated, it might not be so problematic as it first appears. ‘Generation Z’ were born into an online world and have grown up with continuous digital updates [14]. Their adaptivity to online modes of education could be far better than those who preceded them. They are at ease with multi-tasking across devices and they tend to be comfortable with educating themselves via online videos and engaging with peers through typed text, over face-to-face communication [15]. Promisingly, better attendance, engagement, and feedback has been reported in the veterinary setting already [16] and students report that online education is more convenient, time-saving, and flexible for them [6]. Despite this, clear boundaries need to be fashioned by students to separate their online social space with the online learning environment, which may require discipline and maturity beyond their years for the youngest students. Luckily, engagement with online education material can be closely monitored by educators, where legally appropriate, and interventions can be applied quickly.

Video lectures are largely well received by students [17]. They can be recorded and delivered asynchronously which allows greater convenience, flexibility, and control over the learning for the student. Although for some students, having the structured schedule of live, synchronous lectures may be beneficial for managing a sustainable workload [18]. Visual teacher presence, whether live or not, in video content is anecdotally somewhat important. Students appreciate time for online chat at the end of the session (again, whether a pre-recorded or live session) to discuss topics and ask questions. Other feedback has indicated that using storytelling and personal anecdotes, jokes, keeping the session light-hearted, and including popular culture references are all appreciated.

Video lectures need to be balanced within the program as part of multimodal delivery of information, in order to maintain interest [19]. Ranasinghe and Wright [18] suggest that pre-recorded, asynchronous video and live, synchronous lectures could be complementary, with live lectures used to deliver new concepts and pre-recorded video for revision of difficult topics, or to connect with other content or activities (Figure 1). Brockfeld and colleagues [20] demonstrated no difference in the effectiveness of live (in person) lecture versus pre-recorded video in preparing medical students for an exam. However, students may feel more involved and can interact with peers and educators in real time during live teaching, which 48% preferred. Familiarity with the media, integration of videos with other educational materials, and the educational level of students can influence how students perceive the importance of video learning in online learning. As students watch more video content, they became more satisfied with them and gave a higher rating of learning effect [19].

Anecdotally, students have mixed opinions about the length that videos should be for example a one-hour lecture versus four 15-minute ‘chunks’. The optimum is likely to depend on the nature of the content. Longer sessions should still be interspersed with time for activities, interactions and breaks. It is important to signpost students at the beginning of the video what is planned in terms of session structure. When using digital visual and verbal communication tools, educators need to make sure that they do not inadvertently establish extra barriers for those with additional learning needs. Dyslexia-friendly fonts, type size, and backgrounds are easy to use and voice auto recognition for captions is available with some software (e.g., Panopto©^c^, 506 2nd Avenue, Suite 1600, Seattle, WA 98104 USA. https://panopto.com, accessed on 17 June 2021).

Despite the preferred communication styles of this generation of students, encouraging discussion after the delivery of teaching should be encouraged for maximum engagement with the content. Discussion is useful for hypothesising, exploration, debate, as well as idea synthesis [21], and exploratory talk is the most productive type of discussion [22]. With exploratory talk, ideas are challenged but treated with respect as all participants are encouraged to contribute by asking questions and active listening. Ideas are explored by participants contributing to what came before. Educators and moderators of the discussion should make the ground rules explicit before starting the session–encourage everyone to contribute, build an environment of trust and respect, prepare to accept challenge. A discussion icebreaker could be used to start and make it explicit in the language that is used that contributions from all students are valued. Students have expressed worries about virtual delays or clashing with other speakers online, as there are fewer visual clues than during face-to-face discussion. An alternative to this is using the chat box, embedded polling or the raise hand function in the video conferencing software to encourage discussion. 

Specifically relating to covering clinical knowledge content, case discussions are an obvious way to refresh and integrate clinical knowledge. Discussions work best when details of the clinical picture are released to students in stages with tutor facilitated development of clinical reasoning skills along the way. They can include videos of physical examinations, diagnostic images and laboratory results. Mattson [23] has recently described Veterinary Isolated Clinical Education rounds (https://www.youtube.com/channel/UClQbhJjqT_KPD2rv5j82iFQ/about, accessed on 17 June 2021) set up by staff at the University of Florida. It is a volunteer led, crowd sourced continuing education resource that offers video and worksheets to facilitate learning for clinical students. Case based e-learning (CBEL) has been reported in the veterinary education literature to address difficulties with case availability and standardisation. It would be equally suited for clinical teaching during the pandemic where case access is limited. Students reported CBEL tools positively in terms of practicality, interactivity, and accessibility [24], in addition to being highly effective in teaching a methodical approach to a clinical case [24,25]. They may also facilitate students’ transition from problem solving in a well-structured classroom setting to an ill-structured clinical setting [26].

Online peer to peer learning has been reported in the medical field. For example fourth year students volunteering to teach symptom based medicine and surgery topics to third year students, who in turn teach second year students systems-based pathology via video conferencing [27]. This could be easier replicated in the veterinary school, although authors report that their previously cultivated, more informal, in-person atmosphere is difficult to replicate online with the loss of non-verbal cues, and learners are more passive, particularly when cameras are switched off.

#### 2.2.2. The Theoretical Aspects of Online Education

These mitigating approaches have pushed veterinary education further into the virtual world than it has ever been before. However, with their confinement online, educators may ‘deliver’ a course digitally with a lack of awareness of pedagogical shifts in virtual learning environments [28]. The selection of online teaching tools may be based upon availability or familiarity (e.g., pre-recorded or live lectures), but an appreciation of how the tool supports a particular type of content or instruction method is important [29]. Just as no single learning theory can adequately explain teaching in the classroom or workplace, the same is true for online education. Nonetheless, there are several that could be useful to consider as frameworks to strengthen implementation, evidence-based decision making, and scholarly outputs.

Garrison, Anderson, and Archer’s Community of Inquiry supports the use of online (and blended) courses as active learning environments or communities [30,31]. These depend upon instructors and students sharing information and ideas. The model is based on three critical elements for online learning–the social presence, cognitive presence and teaching presence. Social presence is manifested through interaction and indicators include open communication, group cohesion and emotional expression. Cognitive presence is actuated through ‘Practical Inquiry’, a four-step process of a triggering event, idea exploration, integration of knowledge, and resolution, which can be facilitated by critical thinking and reflection. Finally, teaching presence is essential for balancing the cognitive and social elements with the intended learning outcomes–this reflects the anecdotal evidence that students have a preference for seeing and engaging with their teachers in online learning material. The indicators of teaching presence include instructional management, building understanding, and direct instruction. Popular models that align well with a Community of Inquiry which could be utilised as part of a veterinary programme would include use of the flipped classroom, discussion using audio-visual conferencing, discussion boards, and producing wikis.

Siemens, (2004) acknowledges a shift in the way that knowledge flows in the digital era. The internet has moved learning away from an internal and individual activity to a group or community activity [32]. They deviated from more traditional schools of thought such as behaviourism, cognitivism and constructivism when they developed the theory of Connectivism, based on the dynamics of information flow. Siemens theorises that learning can reside outside of the person, within an organisation or database, and focuses on connecting sets of specialised information. Those connections enable learners to learn more and are, in fact, more important than the current state of knowledge. There are eight principles of Connectivism but three to highlight would be that a capacity to know more is more critical than what is currently known, accurate and up to date knowledge is the intent of all learning activities and decision making itself is a learning process. These suggest that students need to understand, and be provided with, opportunities to navigate and assimilate knowledge in an online environment where information is constantly evolving and renewing. This concept is exercised in the self-directed and problem-based learning, where students need to develop the ability to seek out current and relevant information whilst filtering that which is extraneous or irrelevant, often using online sources [33].

Online collaborative learning (OCL) theory from Harasim, (2017) has roots in social constructivism and was developed from text-based asynchronous discussion forums [34]. However, it can be applied to more modern synchronous and audiovisual formats. OCL focuses on how the internet can provide an environment for collaboration and building knowledge. Teaching is operationalised through facilitation as well as being a member of the community. Teachers encourage students to solve problems through discussion, as opposed to rote memorisation of facts. There are three phases to knowledge construction through collaboration: idea generating, idea organising and intellectual convergence. The end result is a final position on the problem, but the learner is never truly finished developing ideas. The strength of this model is that it encourages social interaction amongst learners which is particularly beneficial when students are physically remote, and the development of high-level skills such as critical thinking and idea synthesis which are important skills for veterinary students to gain. A significant weakness is that, with the requirement of a facilitator/moderator for each group of students, it can be challenging to scale up. This model differs from other traditional online courses where information is delivered by students reading or watching, the discussion itself is the core component of teaching. Unlike problem-based learning, OCL also lacks an information gathering stage (from external sources) after idea generation, which could be challenging for veterinary students studying novel concepts.

There are parallels between OCL theory and Instructional Design Theory for Creating Online Learning Communities for Adults from Snyder (2009) [29]. Alongside constructivism, this framework also originates from adult learning theory (particularly Knowles’ andragogy [35]) and learning communities. Similar to OCL, the goal of Snyder’s Online Learning Communities theory is to foster the sharing of information, skills and knowledge among adults with common interests, through online communication, collaboration and interaction. The theory’s guiding principles could be transferred to the design of an online learning community of veterinary student. Specifically, it requires (1) cultivation of a learner centered environment; (2) leveraging of community synergy—improving the quality of learning for all by sharing information, knowledge, skills, experiences, and best practices; (3) respecting individuality, diversity, and experience—each adult is a contributor; (4) focusing on real life problems and (5) promoting self-directed learning.

**Figure 2:**
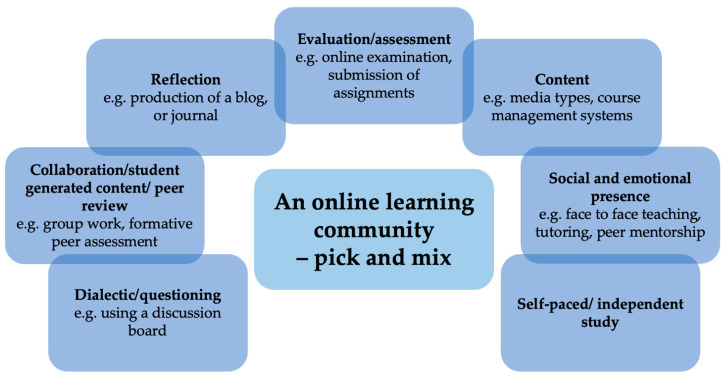
Multimodal Model for Online Education modified from Picciano, (2017) [37].

In an expansion of their Blending With Pedagogical Purpose Model [36], Picciano has developed the Multimodal Model for Online Education [37]. With a focus on a blended program, the approaches used in the model are driven by the pedagogical objectives of the system in which it is being used. However, not all of the seven approaches (Figure 2) need to be employed; for example, self-paced/independent study may not be utilised in a teacher-led blended course, but social activities may feature heavily, and the inverse would be applicable to a distance education course. This flexibility and integration of activities in a ‘pick and mix’ fashion could be of great utility for the development of veterinary programs tailored to each phase of the pandemic and relative stages of restrictions. Additionally, using multiple modalities (both online and in person) will appeal to a wide range of students with different learning needs.

Activities of an online learning community are driven by the pedagogical objectives and the needs of the program. Not all activities are required in all programs.

By using the frameworks and models described and putting online learning theory to work, educators can use these tools to design their own solutions for the individual problems and restrictions imposed upon them in the Covid-19 pandemic and in any local crisis management they may face in the future.

## 3. Practical and Clinical Skills Education, and Workplace Learning

### 3.1. The Problems

Workplace learning is centered around authentic immersion in veterinary practice. However, frequent rotations between specialties and veterinary clinics render students potential vectors for Covid-19 virus. In small animal practice, consultation rooms and theatres require staff to work in close and continuous proximity with each other, and similar applies to veterinarians’ cars in large animal practice. Clients present unknown entities with respect to infection risk and patients could be potential fomites for the virus. In spring 2020, teaching hospitals and private practices reduced their services to urgent and emergency cases only, and as non-essential members of the team, the doors to these experiences were closed to students.

The consequences of reduced real-life experience for students could be far reaching. First and foremost, there will be reduced case exposure that is worked up in real-time and with active involvement of the student. This could result in fewer opportunities for students to apply and restructure their knowledge base and develop clinical reasoning skills. Alongside this, it will be difficult to progress with practical skill development in authentic settings and enculturation is essentially impossible to achieve remotely. Although verbal communication skills can be developed with online role play and simulation, non-verbal skills are difficult to use without being face-to-face. The impact of this change could have a damaging effect on exam performance, but more importantly, there are justified concerns that students will not be able to meet the minimum requirements for core competences directed by the Royal College of Veterinary Surgeons (RCVS) [38]. There are theoretical implications that veterinary courses could be extended, and graduation delayed to make up for deficits. Although this has not come to fruition yet, it could result in increasing student debt and pressures on an already stretched workforce, as fewer new graduates join that academic year.

In a survey of final year medical students from thirty-three medical schools across the UK, the impact of Covid-19 on cancelled examinations and assistantship placements significantly affected students’ feeling of preparedness for the transition from student to doctor [39]. Data from the veterinary sector does not exist yet, but we could reflect on the foot and mouth disease (FMD) epidemic in the UK in 2001 when farm and equine clinical placements largely ground to a halt. A survey at the time demonstrated that about 40% of clinical veterinary students (and more than two-thirds of students in the year below) thought that the epidemic had affected their studies) [40]. However, when farm rotations and extra-mural studies (EMS) were cancelled, the students worked for the Department for Environment, Food & Rural Affairs (DEFRA) to aid in the national effort to get FMD under control. Over 80% of students who worked during the peak of the epidemic felt valued and 83% and 63% said they gained practical and people skills, respectively. If possible, we need to find and support the opportunities for clinical veterinary students to still gain skills in these difficult circumstances. As an example, working or volunteering as part of the human Covid-19 vaccination effort may be possible for students, this would present opportunities to practice communication with the public, improve instrument dexterity and handling clinical/sharps waste [41,42]. However, it is acknowledged that this may be challenging in countries where there is strong segregation of the public health and veterinary sectors.

### 3.2. The Mitigating Approaches

**Figure 3:**
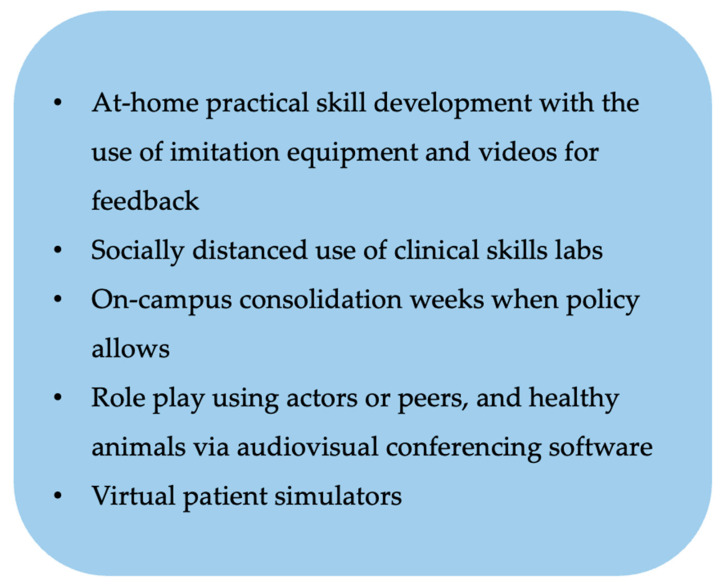
Mitigating approaches for delivering practical and clinical skills content during the Covid-19 pandemic.

Given the proximity required between clinical instructors and students for the supervision of practical tasks involving real patients, in-person clinical teaching may be downgraded to being purely observational. However, the veterinary profession is fortunate that, unlike medical colleagues, veterinary work does not strictly require close human contact to practice many technical skills and so there is still scope for some practical skill acquisition. For obvious reasons, practical skills are difficult to teach remotely, and some creativity will be required to facilitate this. Several options are suggested in Figure 3. Suture kits have been made available for students to take home and practice at University of Wisconsin School of Veterinary Medicine [23]. Students can also video themselves and submit them to the faculty. Other schools are planning for practical consolidation weeks when students return, to consolidate the theory that they are currently learning [16].

Non-technical, clinical skills such as communication and clinical reasoning can be developed to a degree even with social distancing requirements. Effective communication is one of the most crucial components of high-quality veterinary care; it is a core competency highlighted by licensing bodies, veterinarians, and clients alike [43,44,45,46]. Using actors +/− healthy animals, or peers [47], in simulations have been reported to develop veterinary student communication skills [48] and clinical reasoning [49], which could be transferred virtually [50]. Virtual patient simulators have also been evaluated for building medical communication skills [51], mostly involving history taking and delivering bad news. Most simulators have been small sized characters visualised on a computer screen, but immersive virtual environments have also been reported [52]. Virtual communication skills training does have an obvious limitation in terms of building some non-verbal communication skills, and it is labour heavy to develop.

Hands-on clinical training requirements have been altered under the circumstances by the RCVS, which will ease the pressure for both students and staff [23,53]. In the UK, the EMS requirement has been halved for new final year students and they will be able to graduate provided that they are able to demonstrate “that they have achieved all the RCVS Day One Competences”. However normal participation in placements and examinations are expected by licensing bodies elsewhere.

By pooling resources for maximum impact, interprofessional education could lend itself well to the current landscape. Interprofessional education has received little attention in veterinary education even though the importance of the One Health concept is well established [54,55] and veterinary, nursing and other para-professionals work closely together in practice [56,57]. The Covid-19 pandemic could be an ideal opportunity to drive forward with interprofessional education. Additionally, interschool collaboration, drawing from educators across the country will escalate the resource bank rapidly, covering the full curriculum, which no individual institution could easily create and implement quickly [58].

## 4. Assessment

### 4.1. The Problems

In the UK, the RCVS recognises that social distancing has meant that it is necessary to adjust the assessment of veterinary students in some areas. Traditional assessment methods such as written papers in exam halls and objective structured clinical examinations (OSCE) are no longer practical for large groups of students. Additionally, workplace assessments are challenging as clinician assessors face their own struggles with providing a service to clients and patients. Traditional assessments have been honed and validated in the literature, and it is a step onto unsure footing to suddenly change the methods without proper validation in place. Any alternative assessment methods must continue to be “robust, valid and reliable in providing assurance that Day One Competences have been achieved by students upon graduation” [59]. It is also a huge administrative task to organise the infrastructure to be able to deal with the change for both assessments and also graduation ceremonies [60].

### 4.2. The Mitigating Approaches

Fuller and colleagues support the application of a ‘why, who, when, and what’ framework to aid educators in making wise choices about their assessments during the pandemic (Table 1) [11].

Assessment can be delivered online using a secure platform [16,39] and there is the potential to use online proctoring systems [63]. The RCVS Council Covid-19 Taskforce has approved requirements for high stakes assessments that are implemented online/remotely [64]. Computer based examinations can reduce exam-related stress in students that find classroom examination stressful [65], which can only be welcomed in the current climate. It is difficult to invigilate individual students in their own homes, but both the question and option order could be randomised for each student in a best effort to prevent them helping each other. In the absence of proctoring, exams are by necessity open book. Questions should be written with this in mind, not easily answerable using the internet, and used to test higher-order thinking, knowledge application, and problem solving [11]. Bowen reflects that this mirrors real life more closely and that clinical reasoning and problem solving should be the priority over the recall of discrete facts [16]. The technology and network must also be robust to cope with the demands of high-volume, synchronous use, and students who are unable to support themselves with this must be given help from veterinary schools.

Post-hoc analysis of examinations is useful when the methods of assessment are less comprehensively validated [66]. Ideally, pass and failure rates and grades would be similar before, during and after the pandemic, however, that is not necessarily expected and will require well-designed objective studies to characterise. Deviation in pass rates will be influenced by the subject, teaching format, assessment format, and ability of both the educator and student to adapt to learning and being assessed online. The results should be approximately normal in distribution, without a large negative or positive skew. When administering open book examinations which are accessible over a longer period of time (for example 24 h), it would be prudent to track the length of time spent on the examination against the result achieved. This can indicate whether the examination assesses students’ ability to scour resources for information or apply knowledge.

Other key performance indicators such as student dropout, time to graduation, withdrawals from examinations, and the number of attempts required to pass examinations are likely to be affected by the pandemic. These might differ between countries or universities charging high tuition fees and those where university education costs much less, or is free.

Some learning outcomes (namely authentic practical, professional and communication skills) cannot be wholly evaluated with current online tools. Veterinary educators have commenced adapted in-person assessments with appropriate distancing and/or personal protective equipment precautions [11]. When students are able to access workplace learning, a move from an OSCE format to in-clinic assessment could help to ensure that assessors are only encountered once by students, as opposed to repeatedly as in the OSCE format [67]. Mini-Clinical Evaluation Exercises (Mini-CEX) for developing and assessing client interactions and Directly Observed Procedural Skills (DOPS) for practical tasks will be useful and assessment can be made in real time by clinical educators in practice [68,69].

Virtual OSCEs to assess history taking, the knowledge of physical examination, problem solving, decision making and communication using ‘breakout rooms’ in video communication software have been reported in the medical field [70]. Students encounter ‘standardised patients’ with an examiner and invigilator present and their verbal responses are transcribed. An identified key to success was the selection of reliable stations that were previously tested. One limitation is that non-verbal skills cannot be evaluated easily, but this method does allow for assessing student’s ability to adapt to aspects of real life, albeit virtual, patient care.

## 5. Conclusions

**Figure 4:**
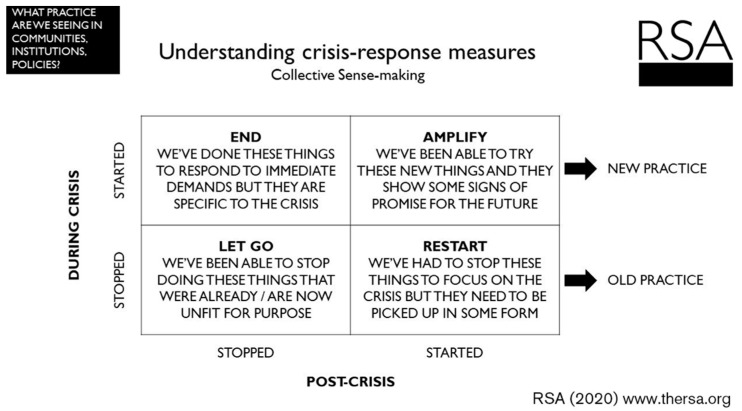
A framework to understand crisis-response measures copied with permission from Royal Society for Arts Manufactures and Commerce, (2020) [71].

The Covid-19 pandemic will inevitably have changed the way that we teach veterinary students in the future, but how so will be elucidated through detailed study which is yet to emerge from the veterinary education research community. Reflecting on the challenge of educating in a global pandemic and the crisis-response measures employed will offer us valuable insights and a vehicle for looking forward. Utilising the framework in Figure 4 [71], we can determine which teaching practices have endured over the years simply because the sector has not had the real impetus to explore other options and can be left behind (‘LET GO’)? Can we appreciate which practices in both teaching and assessment were missed when abandoned by necessity (‘RESTART’)? Finally, and importantly, the innovation and creativity that comes hand in hand with sudden and challenging obstacles will undoubtedly gift us with novel and exciting practices that we can continue to use once the dust has settled (‘AMPLIFY’).

## Figures and Tables

**Table 1:** Rethinking the why, who, when and what of assessment in the face of the Covid-19 pandemic adapted from Fuller et al. (2020) [11].

Rethinking …	Points to Consider
… why we are performing assessment?	Are we performing assessment to determine competency upon graduation? Or the level of student understanding?
… who are we assessing?	Consider the academic stage of the students. This will enable a better understanding of the short- and long-term consequences of changes to assessment. Students in earlier years have more room for manoeuvre and later assessment could be considered, but for students facing licensure high stakes assessments will need to be maintained in some form to validate competency.
… when should we assess?	Considering the intentional and unintentional consequences of timing alterations can aid decision making. For example, as students and staff wrestle with universal change, direct substitution of an assessment at short notice comes with a risk of error as those involved were prepared for a different format. Ask the questions: do we need to assess now?–can it be postponed?
… what type of assessment should we use?	We have the opportunities to consider new types of assessment such as computational adaptive testing [61] or sequential testing [62] which could utilise existing tools and resources in a safe, socially distanced way. Alternatively, a return to more traditional assessment formats such as the viva voce (held virtually or socially distanced) or a well–designed open book examination lend themselves well to clinical cases and problem solving.

## Data Availability

No new data were created or analysed in this study. Data sharing is not applicable to this article.
